# *Tokorhabditis Tauri* n. Sp. and *T. Atripennis* n. Sp. (Rhabditida: Rhabditidae), Isolated from *Onthophagus* Dung Beetles (Coleoptera: Scarabaeidae) from the Eastern USA and Japan

**DOI:** 10.2478/jofnem-2022-0028

**Published:** 2022-08-21

**Authors:** Erik J. Ragsdale, Natsumi Kanzaki, Tatsuya Yamashita, Ryoji Shinya

**Affiliations:** 1 Department of Biology, Indiana University, Bloomington, IN 47405 United States; 2 Kansai Research Center, Forestry and Forest Products Research Institute (FFPRI), Kyoto, Kyoto 612-0855, Japan; 3 School of Agriculture, Meiji University, Kawasaki, Kanagawa 214-8571, Japan

**Keywords:** insect–nematode interactions, morphometrics, new species, taxonomy, trioecy, vivipary

## Abstract

Two new species of *Tokorhabditis*, *T. tauri* n. sp. and *T. atripennis* n. sp., which were isolated from multiple *Onthophagus* species in North America and from *O. atripennis* in Japan, respectively, are described. The new species are each diagnosed by characters of the male tail and genitalia, in addition to molecular barcode differences that were previously reported. The description of *T. tauri* n. sp. expands the suite of known nematode associates of *O. taurus*, promoting ecological studies using a beetle that is an experimental model for insect–nematode–microbiota interactions in a semi-natural setting. Furthermore, our description of a third *Tokorhabditis* species, *T. atripennis* n. sp., sets up a comparative model for such ecological interactions, as well as other phenomena as previously described for *T. tufae*, including maternal care through obligate vivipary, the evolution of reproductive mode, and extremophilic living.

Cow dung provides an experimentally tractable case study for the ecological succession of nematode communities, due to the diversity and densities of nematodes its microbes support and to the rapid turnover inherent to dung’s ephemerality (Sachs, 1950; Sudhaus, 1981). Furthermore, dung can be brought into laboratory mesocosms, allowing controlled studies of its nematode populations (Kühne, 1996; Ledón-Rettig et al., 2018). Important for understanding ecological successions in dung is the influence of the nematodes’ dispersal vectors, such as coprophagous beetles and flies, which necessarily affect colonization by the differential affinities that dung-inhabiting nematodes have for potential vector species (Sachs, 1950; Sudhaus et al., 1988; Sudhaus and Kühne, 1989; Kiontke, 1996; Weller et al., 2010). Communities are likely also influenced by insect-driven transformations of dung: for example, several species of beetles of Scarabaeidae and Geotrupidae modify dung as “brood balls” that they provide for their offspring, influencing the microbiota therein (Estes et al., 2013; Schwab et al., 2016). Given a system in which dung beetles and their brood balls can be manipulated in the laboratory, a description of the associated nematode fauna would enable dissection of the ecological interactions between the beetles and their nematode and microbial communities.

The beetle *Onthophagus taurus* (Schreber) has been established as an experimental model for studying the interplay of environment and development, specifically with respect to an evolutionary novelty, head horns, that they display (Kijimoto et al., 2012; Hu et al., 2019). Furthermore, *O. taurus* stands out for the value of its ecosystem services in the cattle industry: this beetle species has been introduced into several geographic locales beyond its native Mediterranean and Central European range, in some cases systematically, as dung burying prevents forage loss, recycles nitrogen, and reduces pest incidence (Edwards, 2007; Losey and Vaughan, 2008). Toward an understanding of how dung-inhabiting nematodes influence the beetles’ ecology, a previous study identified *Diplogastrellus monhysteroides* (Bütschli, 1876) Paramonov, 1952 (Family Diplogastridae) as a sexually transmitted associate that enhanced the beetles’ fitness during development (Ledón-Rettig et al., 2018). This effect was correlated with the nematodes’ modification of microbial and fungal communities in the brood ball. Further investigation of *O. taurus* from wild-caught populations and laboratory colonies has consistently revealed the presence of another nematode species, since then referred to as *Tokorhabditis* sp. EJR13. As a species of nondiplogastrid “Rhabditidae,” these nematodes likely belong to a different trophic guild than *D. monhysteroides* (Sudhaus, 1981; Yeates et al., 1993). Thus, their impacts on beetle fitness and local microbial communities are likely to differ from, and perhaps interact with, that of the latter species, making *Tokorhabditis* sp. appropriate to consider in future studies of nematode-mediated effects on *O. taurus* and its microbiota.

The genus *Tokorhabditis* (Kanzaki et al., 2021) was recently introduced to describe the extremophilic nematode *T. tufae*, which was collected from an arsenic-rich, alkaline, and hypersaline habitat in Mono Lake, California (Shih et al., 2019). Unlike members of the genus closest to *Tokorhabditis*, *Auanema* (Kanzaki et al. 2017), *T. tufae* shows obligate vivipary, including embryonic growth *in utero* (Kanzaki et al., 2021). Vivipary in rhabditid nematodes is typically facultative, either manifesting as *endotokia matricida* (“bagging”; also “aparity,” *sensu* Sudhaus, 1976) or as live birth of young that hatch from rigid eggs *in utero*. Such cases have been speculated to protect offspring from especially harsh or complex environments, often as a plastic response (Johnigk and Ehlers, 1999; Chen and Caswell-Chen, 2004; Vigne et al., 2021). Although the sediments of Mono Lake present obvious physiological stresses, the habitat of dung may also present an unusually stressful environment to nematodes, and species of Rhabditidae have long been observed to show facultative vivipary in such habitats (Sudhaus, 1976). In *Tokorhabditis*, vivipary has taken on an extreme form, which among Rhabditidae *sensu lato* (i.e., including Diplogastridae; Kiontke et al., 2007) has only been observed, to our knowledge, in the diplogastrid genus *Sudhausia* (Herrmann et al., 2013; Kanzaki et al., 2017). Like the two species we describe below, *Sudhausia* nematodes are, whether coincidentally or causally, associated with *Onthophagus* dung beetles.

In addition to sampling *O. taurus* from two North American locales, collections of *Onthophagus* beetles in Japan revealed a third putative species of *Tokorhabditis*, heretofore referred to as NKZ329. With the discovery of this new species, we are given the ability to make phylogenetic comparisons within *Tokorhabditis*. Furthermore, EJR13 and NKZ329 offer new comparators to *Auanema*, species of which, as in *Tokorhabditis*, are trioecious (with males, females, and self-fertile hermaphrodites) and therefore used as a model for the evolution of reproductive mode (Chaudhuri et al., 2011). To continue building a comparative model for reproductive strategies, maternal care, extremophilic living, and nematode-mediated, host–microbiome interactions, we describe the two new species of *Tokorhabditis* here.

## Materials and Methods

### Nematode collection and culturing

Nematodes of the two new species were obtained from adults of *Onthophagus* spp. In the case of *O. taurus*, nematodes were scraped from the underside of the beetles’ elytra into M9 buffer. For *Onthophagus* sp. collected in Tsukuba, Japan, an adult beetle was dissected on an agar plate (2.0% agar without nutrients) and then kept at room temperature to allow phoretic nematodes to propagate, feeding on microbes originating from the dissected insect cadaver. For both new species, nematodes were cultured on nematode growth medium (NGM) seeded with a lawn of *Escherichia coli* strain OP50, whereupon strains were kept under the culture codes EJR13 and NKZ329. Molecular profiles for the two species were previously phylogenetically analyzed and published (Kanzaki et al., 2021). Specifically, sequences had been obtained for the 18S rRNA gene and deposited in the GenBank database under accession numbers LC639822 and LC639823 for EJR13 and NKZ329, respectively.

### Light microscopic observation and preparation of type specimens

Adult nematodes were collected from 1-week-old cultures, after which they were heat-killed and fixed in TAF (triethanolamine:formalin:distilled water = 2:7:91) for 1 week. Fixed material was processed to glycerin using a modified Seinhorst’s method (Minagawa and Mizukubo, 1994) and mounted in glycerin according to the methods of de Maeseneer & d’Herde (1963). Mounted specimens were used for morphometrics and kept as type material. In addition, live adults from 1-week-old cultures were used for detailed morphological observations following the methods of Kanzaki (2013). All micrographs were obtained using a digital camera system (MC170 HD; Leica, Wetzlar, Germany) and morphological drawings were made using a drawing tube connected to the microscope (Eclipse Ni; Nikon, Tokyo, Japan).

### Scanning electron microscopy (SEM)

To obtain adult males for SEM, two to three dauer juveniles were transferred onto each of several NGM plates seeded with *E. coli* OP50 and incubated at 20ºC, following the protocol of Kanzaki et al. (2021). As for *T. tufae*, males of the new species were present in the next generation at relatively high frequency and were collected into M9 buffer. Males were killed by heating on a hot plate (60ºC for 5 min) and were pre-fixed in 2% formaldehyde plus 2.5% glutaraldehyde in M9 buffer for 15 hr at 4ºC. Nematodes were then post-fixed with 1% osmium tetroxide for 1.5 hr and thereafter dehydrated with an ethanol series (25%, 50%, 70%, 80%, 90%, and twice in 99.5% ethanol). Following dehydration, samples were immersed in 100% tert-butyl alcohol twice, for 15 min per immersion, and then dried with a freeze-dryer (JFD-310; JEOL, Tokyo, Japan). Samples were coated with osmium (Yamaguchi et al., 2016) in a sputter coater (HPC-1SW; Vacuum Device, Ibaraki, Japan), followed by observations under a JSM-6700F (JEOL) scanning electron microscope.

## Results

### Characters common to *Tokorhabditis* n. spp.

The two new species and the type species of *Tokorhabditis*, *T. tufae*, are similar in their morphological characters, whereby hermaphrodites, females, and dauer juveniles are nearly identical other than in morphometric variations. Therefore, common typological characters are described first, after which species-specific characters of adult males are described for each new species.

### Adult

Body cylindrical. Cuticle thick with fine annulation; annuli about 1.5 mm to 2.0 mm wide at mid-body. Lateral field present, not distinctive, ridges (alae) absent. Lip region not clearly offset, with six equal-sized sectors, two dorsal sectors, right lateral and subventral sectors close to left lateral and subventral sectors and forming a somewhat triangular stomatal opening. Each lip with a setiform labial sensillum. Four setiform cephalic sensilla present. Amphids with small, oval-shaped pores at level of posterior end of cheilostom. Stoma cylindrical, separated into cheilostom, gymnostom, and stegostom, with relative lengths of approximately 1:2:3, respectively. Cheilostom and gymnostom forming a simple, short cylinder, with junction between anterior and posterior arcade syncytia faintly visible at middle of gymnostom. Prostegostom and mesostegostom a simple cylinder, forming pharyngeal sleeve, and comprising slightly more than half of stomatal tube; metastegostom slightly anisotopic and isomorphic, with two small denticles on each sector, slightly more posterior on dorsal side. Procorpus muscular, cylindrical; metacorpus forming well-developed median bulb, isthmus slender, basal bulb rounded (i.e., not polygonal) with weak, duplex haustrulum posterior to valves. Procorpus plus metacorpus slightly longer than isthmus plus basal bulb. Cardia (pharyngo–intestinal junction) conspicuous. Nerve ring surrounding middle part of isthmus. Excretory pore conspicuous in ventral and lateral views, variable in position among individuals, and mostly overlapping with level of basal bulb. Excretory duct extending slightly anteriad, then reflexing to continue posteriad. Excretory cell observed slightly posterior to excretory pore. Deirid at approximately same level as or slightly posterior to excretory pore.

### Male

Two separate lines of lateral field only sometimes observed, that is, not conspicuous, with visibility depending on specimen preparation. Tail region weakly ventrally curved when killed by heat. Stoma somewhat narrower than in female/hermaphrodite. Testis single, to right of intestine; anterior part ventrally reflexed. Distal third of gonad forming *vas deferens*, either empty or containing small sperm cells. Two subventral glands and one dorsal cloacal (anal) gland visible at level of anterior end of retracted spicules. Spicules paired, arranged as “V” shape in ventral view, often protracted in heat-killed specimens. In lateral view, spicule with weakly developed manubrium; blade widest at just posterior to manubrium, then smoothly tapered to bluntly pointed tip. Gubernaculum short, narrow, slightly ventrally arcuate in lateral view, approximately half of spicule in length; thin, flat extensions of both sides cover the dorsal side of spicule blade; elongate oval in ventral view. Bursa present, open anteriorly, somewhat polygonal, with smooth edges; distal end of bursa deeply notched, forming a rounded flap on each side of a slender tail spike. Eight (three precloacal and five postcloacal) pairs of genital papillae forming short and thick bursal rays, with their arrangements described below for each species. Phasmids pore-like, with ventral openings in a terminal position near tail tip.

### Female and hermaphrodite

Females morphologically indistinguishable from hermaphrodites. Body weakly smoothly and ventrally arcuate when heat-relaxed. Vulva located at mid-body, forming horizontal slit; cuticle around vulva lacking annulations. Two gonads, one extending anteriad from vulva along right side of body, the other extending posteriad from vulva and along left side; both gonads dorsally reflexed. Germ cells arranged in multiple (two to three) rows in distal end of ovary, with transition to single row of well-developed oocytes arranged in proximal half; oocytes nearest oviduct/uterus are most developed in size and most opaque in cell contents. Oviduct not distinct from uterus; spermatheca at boundary between ovary (or, in hermaphrodites, ovotestis) and oviduct/uterus. Spermatheca not clearly separable from rest of reproductive tract, distinguished only by presence of sperm (small, rounded cells) rather than structure of reproductive tract. Uterus a long, thick-walled tube between spermatheca and vulva/vagina and clearly expands when carrying well-developed embryos and juveniles. Dorsal wall of junction of anterior/posterior uterus thickened. Vagina approximately perpendicular to body surface, folded forming “Z” or more complex shape in young adults, although vaginal folding unclear in mature individuals after laying juveniles, and possessing a thick wall, surrounded by tissue of flattened, elongated cells layered longitudinally, constricted by sphincter muscle at vaginal–uterine junction. Young females/hermaphrodites usually carrying a maximum of one embryo in each uterus; in old individuals, many (more than 10) well-developed embryos and juveniles are present in an expanded uterus, rendering other gonadal structures vague. Overmatured female, that is, mature but not having mated, harbors many unfertilized oocytes in uterus/oviduct. Two subventral glands and one dorsal rectal gland observed surrounding intestine– rectum junction and anterior part of rectum. Rectum approximately same as anal body diam. Anus a short horizontal slit at surface; posterior anal lip protrudes slightly in lateral view. Phasmids at approximately one anal body diam. posterior to anus. Tail forming elongate conoid, smoothly tapered to finely elongated conical tip but not filiform.

### Dauer juvenile

Actively moves around on substrate. Body cylindrical, straight or weakly ventrally arcuate when heat-relaxed. Cuticle thin, smooth, coarsely and shallowly annulated, with two lines of conspicuous lateral field. Anterior end dome-shaped, continuous with body. Amphids with oval-shaped openings, conspicuous, at level of posterior end of cheilostom. Labial sensilla sometimes observed as refractive dots, inconspicuous. Initially sheathed in coarsely annulated J2 cuticle; border between J2 cuticle and dauer body transparent. Stoma narrow, cylindrical, weakly sclerotized, anterior end closed; separations among cheilostom, gymnostom, and stegostom not clear, but stegostom distinguished by presence of pharyngeal sleeve. metastegastom and telostegostom, where stoma meets pharyngeal lumen, more sclerotized than anterior regions of stoma. Procorpus cylindrical, not well-developed, occupying less than one-third of corresponding body diam. Metacorpus slightly expanded to form median bulb. Isthmus slightly slenderer than procorpus. Posterior end of pharynx forms weakly developed basal bulb with weakly developed duplex haustrulum, smoothly connected to cardia. Procorpus plus metacorpus (corpus, or anterior pharynx) slightly longer than isthmus plus basal bulb (posterior pharynx). Cardia funnel-shaped, closed. Nerve ring not conspicuous, surrounding middle of isthmus. Excretory pore conspicuous, on ventral side of body, at level of posterior part of isthmus. Excretory tube extending anteriorly, and then reflexing posteriorly. Excretory cells sometimes observed, but not always clear. Genital *anlagen* visible ventrally at mid-body; cells linearly arranged, but number of cells not clearly observed. Rectum approximately same as anal body diam. in length. Tail elongate conoid with bluntly pointed tip.

### Tokorhabditis tauri* n. sp. = Tokorhabditis sp. EJR13 apud Kanzaki et al. (2021).

(Figs. 1–8)

**Figure 1:**
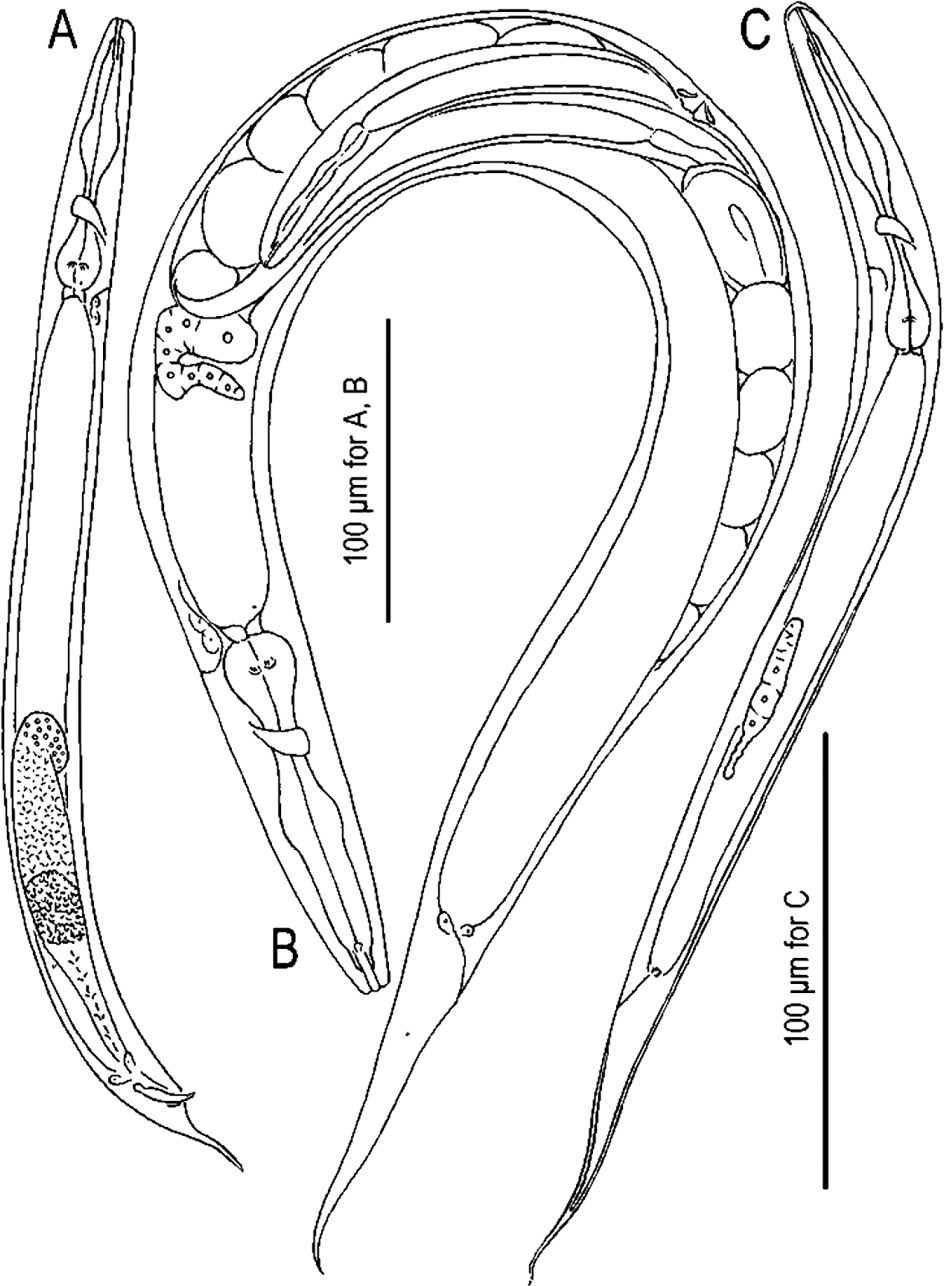
Mature male, hermaphrodite, and dauer juvenile of *Tokorhabditis tauri* n. sp. (A) Male. (B) Mature hermaphrodite. (C) Dauer juvenile.

### Measurements

See Table 1.

**Table 1:** Morphometric values for *Tokorhabditis tauri* n. sp.

	Holotype male	Paratype males	Paratype hermaphrodites	Paratype females	Paratype dauers
N	–	9	10	10	10
L	364	352 ± 36 (309–415)	905 ± 149 (720–1241)	912 ± 80 (741–1021)	286 ± 11 (263–299)
A	15.5	17.5 ± 1.0 (15.5–19.0)	15.0 ± 0.9 (13.8–16.6)	12.6 ± 0.8 (11.5–14.1)	21.0 ± 1.4 (18.5–22.9)
B	4.1	4.3 ± 0.4 (3.9–4.9)	8.0 ± 0.9 (6.6–9.7)	8.6 ± 0.5 (7.3–9.1)	3.8 ± 0.1 (3.5–4.0)
c^a^	13.9	13.1 ± 1.6 (10.6–15.6)	9.3 ± 1.0 (7.9–11.1)	11.9 ± 1.2 (10.4–13.5)	7.1 ± 0.6 (6.5–8.6)
c'^a^	2.3	2.4 ± 0.2 (2.0–2.7)	5.5 ± 0.5 (4.6–6.2)	4.2 ± 0.5 (3.2–5.0)	5.3 ± 0.3 (4.9–6.1)
T^b^ or V	43.4	41.7 ± 2.4 (36.7–45.3)	49.6 ± 1.2 (47.8–52.1)	49.6 ± 1.0 (48.2–51.0)	–
Maximum body diam.^c^	23.4	20.2 ± 2.8 (17.0–24.1)	61 ± 10.3 (43–82)	73 ± 8.8 (53–84)	13.7 ± 0.6 (12.8–14.2)
Stoma diam.	2.1	2.4 ± 0.3 (2.1–2.8)	4.0 ± 0.4 (3.5–5.0)	3.6 ± 0.3 (3.2–4.3)	–
Stoma depth	11.7	11.5 ± 0.7 (9.9–12.4)	14.5 ± 1.5 (12.8–18.4)	15.1 ± 1.1 (13.5–17.0)	–
Stoma depth/diam. ratio	5.5	4.7 ± 0.5 (4.0–5.5)	3.6 ± 0.3 (3.2–4.0)	4.2 ± 0.5 (3.6–5.3)	–
Anterior pharynx length	36	34 ± 2.6 (30–37)	46 ± 3.1 (42–53)	42 ± 1.4 (40–44)	31 ± 0.9 (30–32)
Posterior pharynx length	41	37 ± 2.1 (34–41)	52 ± 3.1 (49–58)	49 ± 3.3 (44–55)	32 ± 1.1 (30–33)
Anterior/posterior pharynx length ratio	0.87	0.92 ± 0.06 (0.86–1.01)	0.89 ± 0.04 (0.83–0.95)	0.86 ± 0.07 (0.74–0.95)	0.98 ± 0.02 (0.95–1.02)
Median bulb diam.	10.6	10.4 ± 0.6 (9.2–11.3)	17.0 ± 2.2 (14.0–21.1)	20.4 ± 1.5 (18.7–22.2)	6.0 ± 0.5 (5.0–6.4)
Basal bulb diam.	14.2	13.2 ± 0.6 (12.4–14.2)	20.4 ± 1.9 (17.5–23.4)	25.3 ± 1.4 (22.2–26.9)	6.9 ± 0.7 (5.7-7.8)
Nerve ring from anterior end	60	56 ± 4.7 (50–64)	79 ± 5.2 (71–90)	72 ± 3.0 (64–74)	50 ± 1.2 (48–51)
Secretory-excretory pore from anterior end	81	78 ± 6.7 (71–90)	113 ± 9.6 (102–131)	106 ± 7.0 (95–115)	58 ± 2.2 (54–61)
Cloacal or anal body diam.	11.3	11.2 ± 0.7 (10.6–12.8)	17.8 ± 2.3 (15.2–22.2)	18.5 ± 0.9 (17.5–19.9)	7.6 ± 0.7 (6.4–8.5)
Tail length^a^	26.2	26.9 ± 1.5 (24.8–29.1)	97 ± 12 (84–119)	77 ± 8.4 (63–90)	40 ± 3.0 (34–43)
Tail spike length	15.6	17.7 ± 1.6 (15.6–21.3)	–	–	–
Whole gonad length	158	147 ± 14 (125–165)	–	–	–
Reflex part of testis	25	19 ± 5.2 (13–27)	–	–	–
*Vas deferens* length^b^	54	56 ± 7.0 (47–70)	–	–	–
*Percentage of vas deferens* to whole gonad	34.1	38.5 ± 3.7 (34.1–46.2)	–	–	–
Spicule length (curve)	17.7	18.3 ± 1.3 (15–6–19.9)	–	–	–
Spicule length (chord)	17.0	17.6 ± 1.3 (14.9–19.1)	–	–	–
Gubernaculum length (chord)	9.2	10.4 ± 0.9 (9.2–12.1)	–	–	–
Anterior ovary length		–	87 ± 15 (67–113)	125 ± 6.4 (117-135)	–
Posterior ovary length		–	77 ± 14 (53–96)	118 ± 10 (104–133)	–
Anterior/posterior ovary length ratio		–	1.15 ± 0.19 (0.91–1.45)	1.06 ± 0.07 (0.92–1.15)	–
Phasmid from anus		–	16.7 ± 1.9 (14.0–19.9)	13.6 ± 1.8 (10.5–16.4)	–
Relative position of phasmid to anal body diam.^d^		–	0.95 ± 0.11 (0.80–1.08)	0.73 ± 0.10 (0.58–0.88)	–
Relative position of phasmid to tail length^e^		–	17.5 ± 2.9 (13.0–21.5)	17.9 ± 2.9 (11.7–21.5)	–

a Tail length including tail spike. ^b^Gonad length including reflexed part and *vas deferens*. ^c^Body diam. is maximum at vulval position in hermaphrodite and female (vulval body diam. = maximum body diam.). ^d^Calculated as anus-phasmid distance/anal body diam. ^e^Calculated as ×100 anus-phasmid distance/whole tail length.

### Male

Condylus club-like, clearly separated from other part of spicule by a constriction. Genital papillae and phasmid arranged as <P1, (P2, P3), CO, (P4, P5d), (P6, phasmid, P7d, P8)>, with CO being the cloacal opening, and where P2 and P3 are close to each other, P4 and P5d are almost at the same level, and P6, P7d, P8 are close to each other.

### Hermaphrodite, female, and dauer juvenile

As described above.

### Type habitat and locality

The new species was isolated, and thereafter brought into culture as EJR13, from an adult of *Onthophagus taurus* collected on 17 April 2015 on a dairy farm in Hillsborough, North Carolina (35° 58' 43.49" N, 79° 8' 51.78" W, 178 m a.s.l.). The new species was again collected from *O. taurus* in the same pasture on 16 May 2022. On both dates, *O. taurus* adults were collected by sifting pats of cow dung, and nematodes were thereafter isolated from beneath the beetles’ elytra.

**Figure 2:**
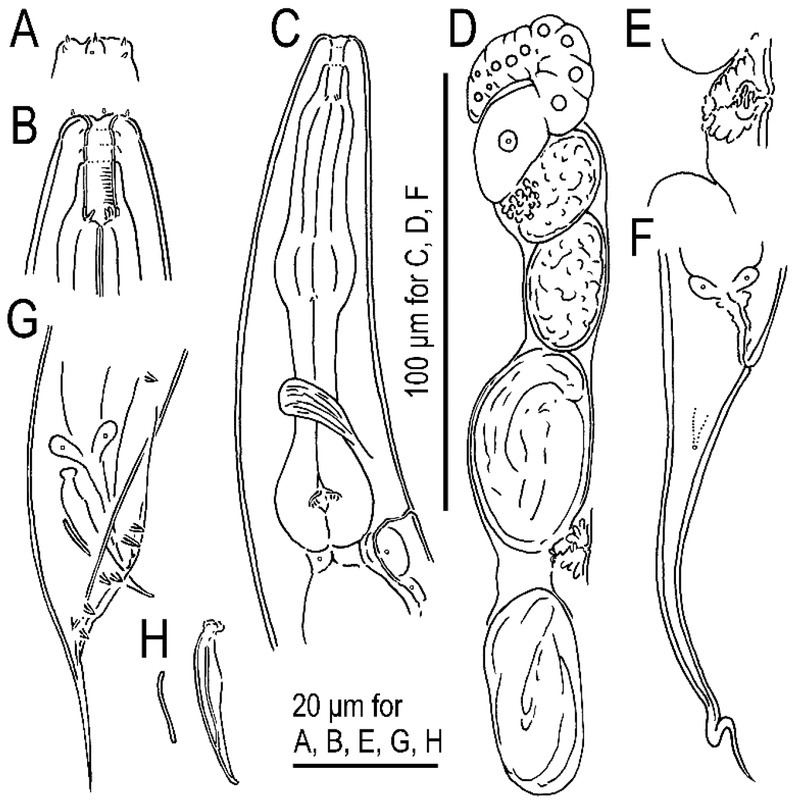
Hermaphrodite and male of *Tokorhabditis tauri* n. sp. All drawings are in right lateral view. (A) Surface of lip region of hermaphrodite. (B) Stomatal region of hermaphrodite. (C) Anterior part of hermaphrodite. (D) Anterior gonad of mature hermaphrodite. (E) Vulval region of young hermaphrodite. (F) Tail region of hermaphrodite. (G) Male tail region. (H) Spicule and gubernaculum.

### Other habitats and localities

The new species was also isolated from *O. taurus* collected, as above, from cow dung on a pasture in Bloomington, Indiana (39° 3' 10.96" N, 86° 36' 9.29" W, 258 m a.s.l.) on 24 April 2015. The species has been frequently found as high-density populations in *O. taurus* brood balls in laboratory *O. taurus* colonies at Indiana University, with the colonies’ source populations being from either of the two sites above (Hillsborough, NC and Bloomington, IN). Because these *O. taurus* colonies used cow dung that was frozen and thawed, a process that kills nematodes originally present in the raw dung (Ledón-Rettig et al., 2018), we rule out dung as the source of nematodes in those brood balls. Therefore, *T. tauri* n. sp. populations in *O. taurus* brood balls were established either through transmission by the beetles themselves or from cross-contamination of other beetle colonies kept in proximity. Additionally, *T. tauri* n. sp. was isolated from *Onthophagus tuberculifrons* Harold caught using fresh cattle dung-baited pitfall traps (Bertone et al., 2005; Kaufman and Wood, 2012) set at the Beef Cattle Teaching Unit at the University of Florida in Gainesville, Florida (29° 37' 19.60" N, 82° 2'14.70" W, 27 m a.s.l.) in October 2015 (“unidentified rhabditid species” in Kanzaki et al., 2017, p. 576). Finally, *T. tauri* n. sp. was isolated from the elytra wild-caught *Digitonthophagus* (= *Onthophagus*) *gazella* (Fabricius) collected in Santa Fe, Florida in May 2022.

### Type material

Type specimens include a holotype male, 9 paratype males, 10 hermaphrodites, 10 paratype females, and 10 dauer juveniles and were deposited as follows.

**Figure 3:**
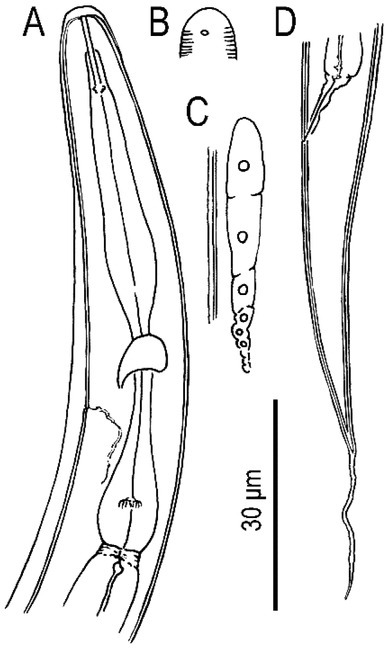
Dauer juvenile of *Tokorhabditis tauri* n. sp. All drawings are in left lateral view. (A) Anterior region. (B) Surface of lip region. (C) Genital *anlagen*. (D) Tail region.

In the USDA Nematode Collection (USDANC), Beltsville, Maryland: the holotype male (T-767t), four paratype males (T-7632p to T-7635p), five paratype hermaphrodites (T-7636p to T-7640p), five paratype females (T-7641p to T-7645p), and five dauer juveniles (T-7646p to T-7650p). In the Swedish Museum of Natural History (SNHM), Stockholm, Sweden: five paratype males (Type-9832 to Type-9386), five paratype hermaphrodites (Type-9387 to Type-9391), five paratype females (Type-9392 to Type-9396), and five dauer juveniles (Type-9397 to Type-9401). In addition, several mounted and unmounted specimens of males, females, hermaphrodites, and dauer juveniles were deposited in the Kansai Research Center, FFPRI. The new species binomial has been registered in the ZooBank database (zoobank.org) under the identifier 693D3EF1-6F91-4469-B8ED-4C652868BDA8.

### Tokorhabditis atripennis* n. sp. = Tokorhabditis sp. NKZ329 apud Kanzaki et al. (2021)

(Figs. 8–14; Supplementary Movie S1)

### Measurements

See Table 2.

**Table 2:** Morphometric values for Tokorhabditis atripennis n. sp.

	Holotype male	Paratype males	Paratype hermaphrodites	Paratype females	Paratype dauers
n	–	9	10	10	10
L	362	362 ± 13 (346–387)	792 ± 32 (734–829)	884 ± 69 (767–966)	278 ± 6.7 (270–292)
a	14.9	14.4 ± 0.6 (13.4–15.2)	14.4 ± 0.7 (13.2–15.4)	11.8 ± 0.9 (10.3–13.2)	20.1 ± 0.7 (19.2–21.1)
B	4.2	4.2 ± 0.1 (4.1–4.3)	7.2 ± 0.2 (6.7–7.6)	8.1 ± 0.5 (7.4–8.9)	3.5 ± 0.1 (3.3–3.6)
c^a^	11.8	13.2 ± 0.9 (11.8–14.7)	6.0 ± 0.3 (5.5–6.3)	6.3 ± 0.5 (5.4–6.9)	7.1 ± 0.3 (6.8–7.6)
c'^a^	2.6	2.3 ± 0.2 (2.0–2.6)	8.4 ± 0.5 (7.5–9.1)	8.0 ± 0.7 (6.9–9.3)	4.8 ± 0.2 (4.5–5.3)
T^b^ or V	41.3	43.6 ± 1.4 (40.8–44.7)	46.2 ± 0.5 (45.4–47.0)	46.3 ± 1.4 (44.6–49.2)	–
Maximum body diam.^c^	24.3	25.1 ± 1.4 (23.4–28.1)	55 ± 3.4 (50–59)	75 ± 9.4 (58–90)	13.8 ± 0.4 (13.3–14.3)
Stoma diam.	2.2	1.8 ± 0.3 (1.4–2.5)	3.2 ± 0.2 (2.9–3.3)	3.2 ± 0.3 (2.6–3.6)	–
Stoma depth	10.8	11.2 ± 0.5 (10.8–11.9)	14.4 ± 0.3 (14.0–14.7)	14.2 ± 0.6 (13.3–15.3)	–
Stoma depth/diam. ratio	5.0	6.2 ± 0.9 (4.7–7.8)	4.5 ± 0.3 (4.2–5.0)	4.4 ± 0.5 (3.9–5.4)	–
Anterior pharynx length	34	35 ± 2.2 (32–38)	51 ± 2.7 (47–56)	44 ± 2.2 (40–47)	34 ± 0.8 (33–36)
Posterior pharynx length	39	39 ± 1.5 (36–41)	48 ± 1.1 (46–50)	49 ± 1.7 (46–51)	33 ± 0.9 (31–34)
Anterior/posterior pharynx length ratio	0.86	0.91 ± 0.06 (0.82–0.99)	1.07 ± 0.04 (1.00–1.17)	0.90 ± 0.06 (0.80–1.02)	1.05 ± 0.05 (1.01–1.17)
Median bulb diam.	10.4	10.7 ± 0.3 (10.4–11.2)	17.6 ± 0.4 (17.3–18.4)	20.3 ± 1.5 (18.4–22.4)	6.4 ± 0.3 (5.9–6.8)
Basal bulb diam.	14.0	13.9 ± 0.4 (13.3–14.7)	20.0 ± 0.7 (18.8–21.0)	24.4 ± 1.3 (21.9–26.0)	7.4 ± 0.2 (7.0–7.7)
Nerve ring from anterior end	59	57 ± 2.5 (53–60)	77 ± 3.2 (72–83)	72 ± 3.3 (66–76)	52 ± 1.1 (50–54)
Secretory-excretory pore from anterior end	80	79 ± 1.8 (77–82)	103 ± 3.5 (99–111)	103 ± 4.1 (94–107)	62 ± 2.6 (58–68)
Cloacal or anal body diam.	11.9	11.9 ± 0.5 (11.2–12.6)	15.8 ± 0.7 (14.7–16.9)	17.8 ± 1.6 (14.8–19.9)	8.2 ± 0.4 (7.6–8.7)
Tail length^a^	30.6	27.5 ± 1.7 (24.8–30.6)	132 ± 5.0 (124–141)	142 ± 11.5 (126–158)	39 ± 1.4 (37–42)
Tail spike length	18.7	16.7 ± 2.0 (13.7–19.4)	–	–	–
Whole gonad length^b^	150	157 ± 7.5 (150–171)	–	–	–
Reflex part of testis	17	19 ± 2.4 (15–23)	–	–	–
*Vas deferens* length	76	76 ± 7.3 (61–88)	–	–	–
*Percentage of vas deferens* to whole gonad	50.6	48.2 ± 3.4 (41.0–53.0)	–	–	–
Spicule length (curve)	21.2	21.5 ± 1.1 (20.1–23.4)	–	–	–
Spicule length (chord)	19.8	20.4 ± 1.0 (19.1–21.6)	–	–	–
Gubernaculum length (chord)	11.2	11.2 ± 0.7 (10.1–12.2)	–	–	–
Anterior ovary length	–	–	104 ± 11 (94–126)	126 ± 19 (100–158)	–
Posterior ovary length	–	–	111 ± 10 (97–125)	132 ± 21 (94–161)	–
Anterior/posterior ovary length ratio	–	–	0.94 ± 0.07 (0.81–1.04)	0.96 ± 0.11 (0.72–1.08)	–
Phasmid from anus	–	–	17.2 ± 1.1 (15.5–19.1)	15.3 ± 2.3 (11.2–18.9)	–
Relative position of phasmid to anal body diam.^d^	–	–	1.09 ± 0.10 (0.98–1.25)	0.86 ± 0.12 (0.76–1.09)	–
Relative position of phasmid to tail length^e^	–	–	13.0 ± 1.1 (11.4–14.5)	10.9 ± 1.9 (8.7–15.0)	–

a Tail length including tail spike. ^b^Gonad length including reflexed part and *vas deferens*. ^c^Body diam. is maximum at vulval position in hermaphrodite and female (vulval body diam. = maximum body diam.). ^d^Calculated as anus-phasmid distance/anal body diam. ^e^Calculated as ×100 anus-phasmid distance/whole tail length.

### Male

Condylus not well-developed, squared, unclearly separated from the other part of spicule. Genital papillae and phasmid arranged as <P1, P2, P3, CO, (P4, P5d), P6, (phasmid, P7d, P8)>, where P2, P3, P4+P5d, P6, P7d+P8 are equally spaced along tail.

### Hermaphrodite, female, and dauer juvenile

As described above.

### Type habitat and locality

This species was originally isolated, and thereafter brought into culture as NKZ329, from *Onthophagus* sp. collected in an experimental stand at the Forestry and Forest Products Research Institute (FFPRI), Tsukuba, Japan on 23 June 2014 (36° 00' 33" N, 140° 07' 37" E, 23 m a.s.l.).

### Other habitats and localities

Since the original isolation of NKZ329, *T. atripennis* n. sp. has been repeatedly isolated from adults of *Onthophagus (Gibbonthophagus) atripennis* Waterhouse collected at the Kansai Research Center, FFPRI, Kyoto, Japan; the new species was also isolated from *O. atripennis* collected at the Ikuta campus of Meiji University, Kawasaki, Japan (35° 36' 39.8" N, 139° 32' 55.8" E, 70 m a.s.l.) and at the Kurokawa Field Science Center, Kawasaki, Japan (35° 36' 31.5" N, 139° 27' 20.8" E, 111 m a.s.l.).

### Type material

Type specimens include a holotype male, 9 paratype males, 10 hermaphrodites, 10 paratype females, and 10 dauer juveniles and were deposited as follows. In the USDANC: the holotype male (T-768t), four paratype males (T-7651p to T-7654p), five paratype hermaphrodites (T-7655p to T-7659p), five paratype females (T-7660p to T-7664p), and five dauer juveniles (T-7665p to T-7669p). In the SMNH: five paratype males (Type-9402 to Type-9406), five paratype hermaphrodites (Type-9407 to Type-9411), five paratype females (Type-9412 to Type-9416), and five dauer juveniles (Type-9417 to Type-9421). In addition, several mounted and unmounted specimens of males, females, hermaphrodites, and dauer juveniles were deposited in the Kansai Research Center, FFPRI. The new species binomial has been registered in ZooBank under the identifier 693D3EF1-6F91-4469-B8ED-4C652868BDA8.

**Figure 4:**
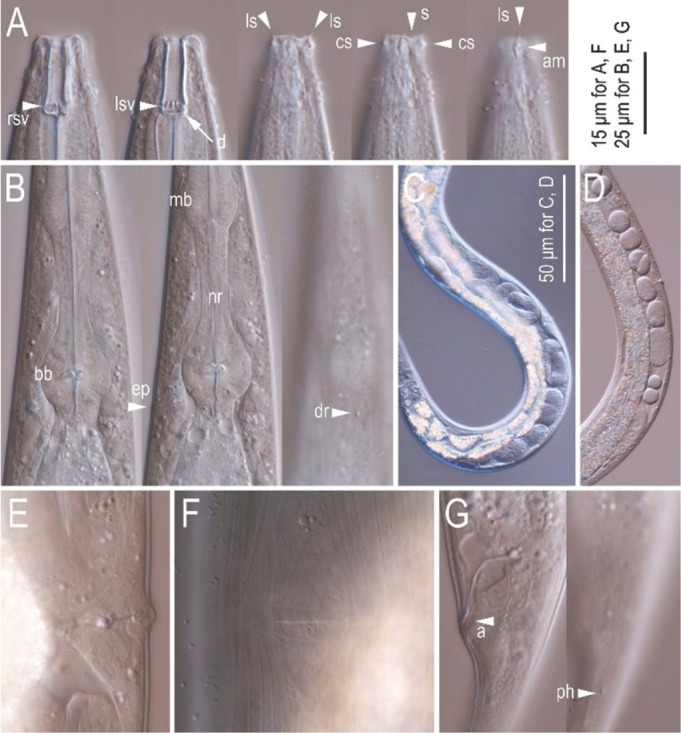
Differential interference contrast micrographs of the hermaphrodite and female of *Tokorhabditis tauri* n. sp. All imaged individuals are of hermaphrodite except for (D), showing female. (A) Lip and stomatal region in left lateral view in five focal planes. (B) Posterior pharynx region in left lateral view in three focal planes. (C) Entire gonad of mature hermaphrodite in right lateral view. (D) Gonadal region of overmature female in right lateral view. (E), (F). Vulval region of young hermaphrodite in right lateral (E) and ventral (F) views. (G). Anal region in left lateral view in two focal planes. a, anus; am, amphid; bb, basal bulb; cs, cephalic sensilla; dr, deirid; d, dorsal denticles; ep, excretory pore; ls, labial sensilla; lsv, left subventral denticles; mb, median bulb; nr, nerve ring; ph, phasmid; rsv, right subventral denticles.

## Diagnosis and relationships

*Tokorhabditis* contains three species, including the two species described here. The new species are morphologically similar to each other and to the type species, *T. tufae*, but can each be diagnosed by characters of the male tail and genitalia. First, *T. tauri* n. sp. is characterized by a square-shaped manubrium separated from the rest of spicule by a clear constriction, whereas *T. atripennis* n. sp. is characterized by a less-developed manubrium without a clear constriction. Second, *T. tauri* n. sp. is characterized by its genital papillae being arranged as <P1, (P2, P3), CO, (P4, P5d), (P6, phasmid, P7d, P8)>, where P2 and P3 are close to each other, P4 and P5d are almost at the same level, and P6, P7d, and P8 are close to each other; by contrast, *T. atripennis* n. sp. is characterized by an arrangement of genital papillae as <P1, P2, P3, CO, (P4, P5d), P6, (phasmid, P7d, P8)>, where P2, P3, P4 + P5d, P6, P7d + P8 are equally spaced from each other.

**Figure 5:**
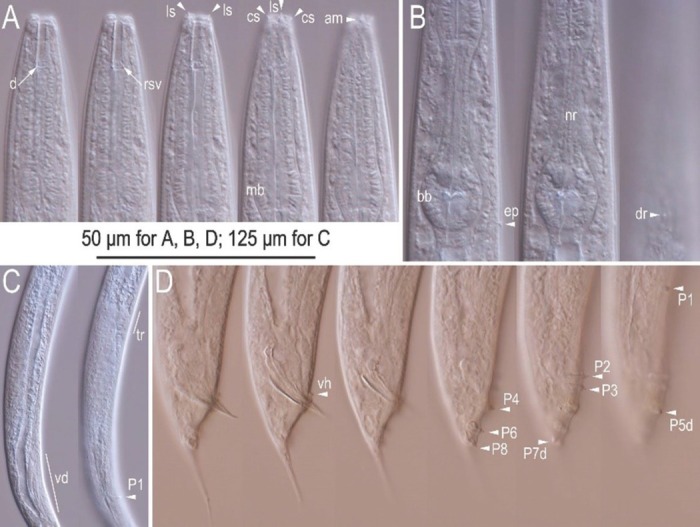
Differential interference contrast micrographs of the adult male of *Tokorhabditis tauri* n. sp. All images are in right lateral view. (A) Lip to anterior pharynx region in five focal planes. (B) Posterior pharynx region in three focal planes. (C) Entire gonad in two focal planes. (D) Tail region in six focal planes. Genital papillae are labeled with the prefix “P”; suffix “d” indicates papillae that open dorsally or laterally. am, amphid; bb, basal bulb; cs, cephalic sensilla; d, dorsal denticles; dr, deirid; ep, excretory pore; ls, labial sensilla; nr, nerve ring; tr, testis reflection; vd, *vas deferens*; vh, ventral precloacal hook.

The two new species are readily distinguished from *T. tufae* by the arrangements of their genital papillae: whereas all papillae form short and thick bursal rays in *T. tauri* n. sp. and *T. atripennis* n. sp., the first four pairs form two doublets of papilliform papillae in *T. tufae*.

Additionally distinguishing *T. tauri* n. sp. from *T. atripennis* n. sp. and *T. tufae* are 7 and 10 nucleotide differences, respectively, in an 846-bp fragment of its 18S rRNA gene; *T. atripennis* n. sp. is distinguished from *T. tufae* by 10 differences in the same 846-bp gene fragment.

The two new species also show some similarities in male tail characters to species of *Rhabditoides* Goodey, 1929 *sensu* Sudhaus (2011), a genus characterized by a narrow, trace-like leptoderan bursa and anteriorly located v1 paired papillae. Although *Rhabditoides* species typically have nine pairs of genital papillae, species with eight pairs in an arrangement like that of the two new species have been reported, such as *R. saprophilus* (Gagarin, 2000), which has an arrangement of <P1, P2, P3, CO, (P4, P5d), P6, (phasmid, P7d, P8)> (Gagarin, 2000). However, the males of all *Tokorhabditis* species, including the two new species, are distinguished from *Rhabditoides* species by not having long, bristle-like labial sensilla, which are a diagnostic character of the latter genus (Scholze and Sudhaus, 2011). Beyond these differences, the character that most clearly diagnoses all *Tokorhabditis* from *Rhabditoides* species, as well as from most or all other species of “Rhabditidae” outside of Diplogastridae (Sudhaus, 2011), is the obligate live-bearing of larval offspring, which are always seen in the uteri of older, mated females and hermaphrodites.

**Figure 6:**
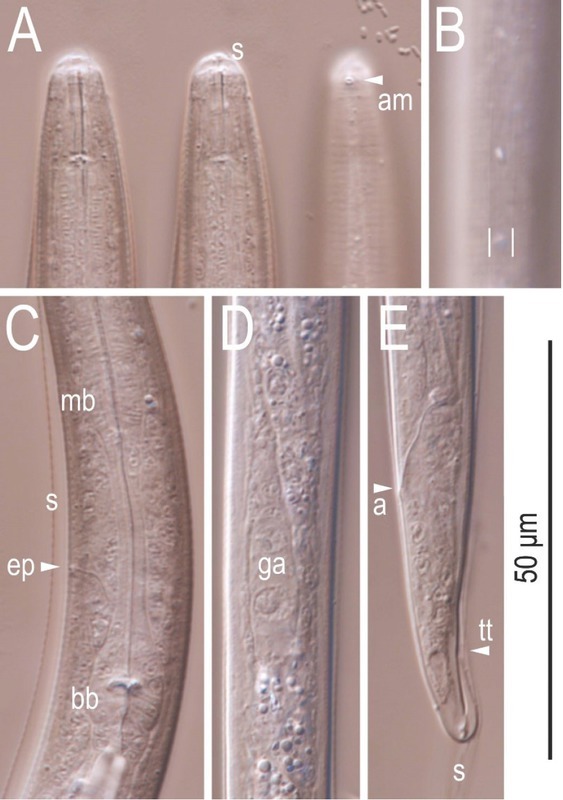
Differential interference contrast micrographs of the dauer juvenile of *Tokorhabditis tauri* n. sp. All images are in left lateral view. (A) Lip and stomatal region in three focal planes. (B) Surface structure of mid-body. Parallel lines mark lateral lines. (C) Middle to posterior part of pharynx. (D) Genital *anlage*. (E) Tail region. a, anus; am, amphid; bb, basal bulb; ga, genital *anlage*; ep, excretory pore; mb, median bulb; s, sheath; tt, tail tip.

**Figure 7:**
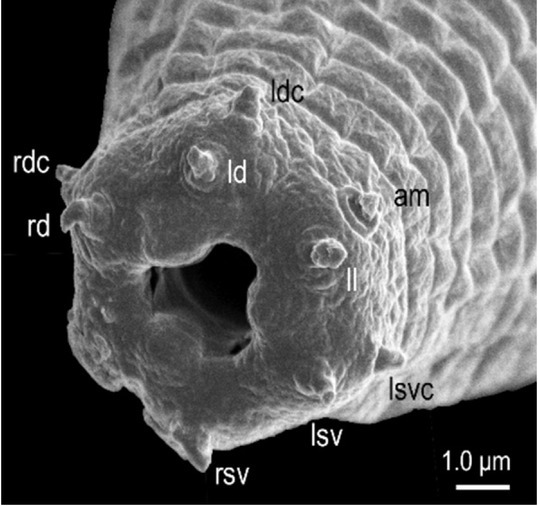
Scanning electron micrograph of the labial region of a female of *Tokorhabditis tauri* n. sp. am, amphid; ld, left dorsal labial sensillum; ldc, left dorsal cephalic sensillum; ll, left lateral labial sensillum; lsv, left subventral labial sensillum; lsvc, left subventral cephalic sensillum; rd, right dorsal labial sensillum; rdc, right dorsal cephalic sensillum; rsv, right subventral labial sensillum.

**Figure 8:**
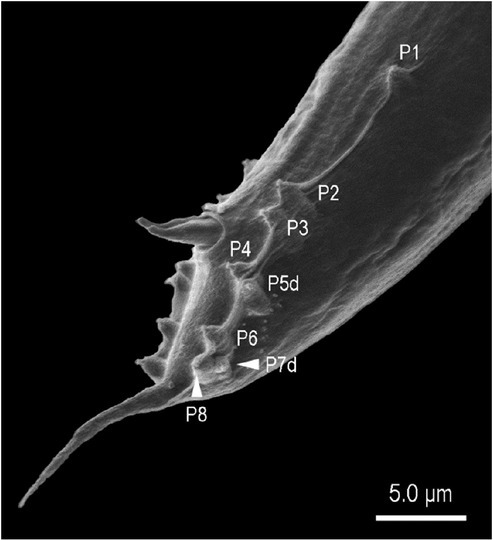
Scanning electron micrograph of the male tail of *Tokorhabditis tauri* n. sp. Genital papillae are labeled with the prefix “P”; suffix “d” indicates papillae that open dorsally or laterally.

## Discussion

Our description of *T. tauri* n. sp. and *T. atripennis* n. sp. reports a new clade of “Rhabditidae” associated with *Onthophagus* dung beetles. In his survey of *Onthophagus* sp. from the Franconia region of Germany, Sachs (1950) identified several nematode associates, which were phoretic under the elytra or in the folds between abdominal segments. These species all belonged to Rhabditidae *sensu lato*, specifically the genus *Pelodera* (*P. cylindrica*, *P. tretzeli*, and *P. voelki*) and the two diplogastrid morphospecies *Fictor stercorarius* and *Paroigolaimella coprophages*; of these, *P. tretzeli* (another viviparous species, albeit without embryonic growth) and *Fictor stercorarius* were the most common. In addition to these species, *Diplogastrellus monhysteroides* has since been found to associate with *O. taurus*, being transmitted through the latter’s genitalia (Ledón-Rettig et al., 2018). All the above *Onthophagus* associates belong to a wider set of mostly rhabditid (including diplogastrid) species repeatedly found to form dung–nematode communities in Central Europe (Paesler, 1946; Sachs, 1950; Sudhaus, 1981; Sudhaus et al., 1988; Kühne, 1996; Susoy et al., 2015). Here, we have identified two new species of *Onthophagus* associates through our sampling of beetles from locales outside of this geographic range.

**Figure 9:**
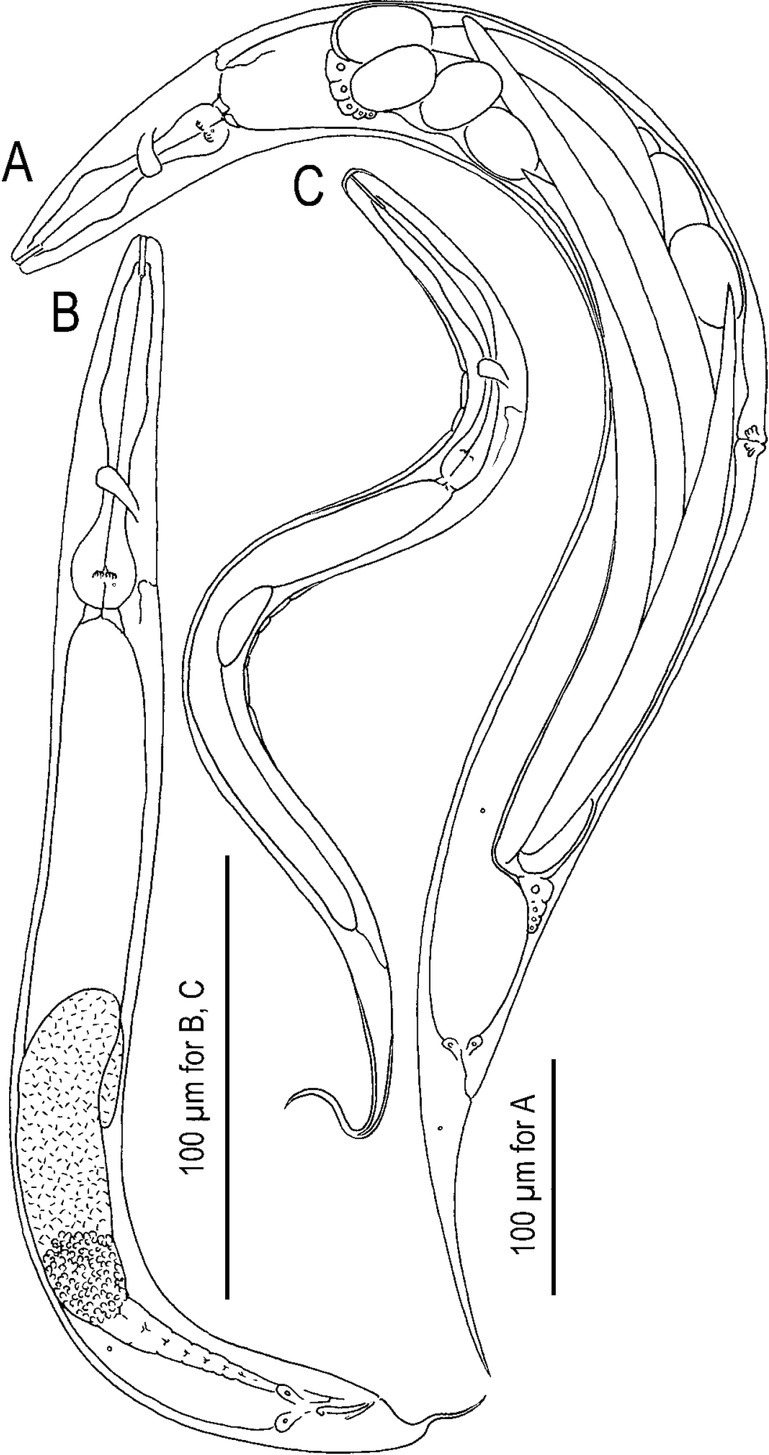
Mature hermaphrodite, male, and dauer juvenile of *Tokorhabditis atripennis* n. sp. (A) Mature hermaphrodite. (B) Male. (C) Dauer juvenile.

**Figure 10:**
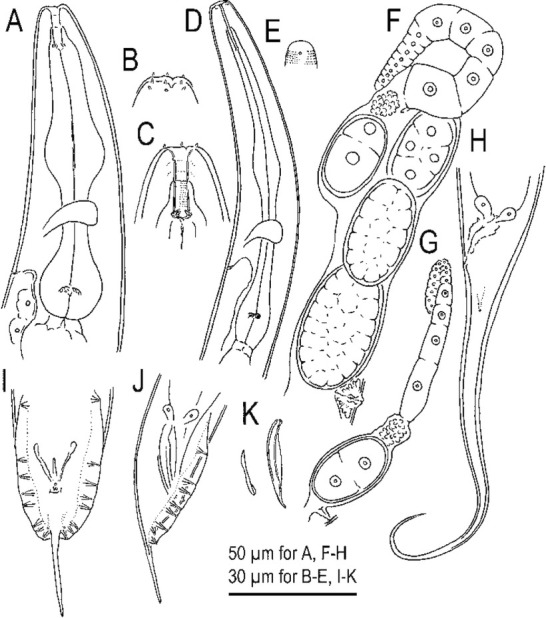
Hermaphrodite, male and dauer juvenile of *Tokorhabditis atripennis* n. sp. (A) Anterior part of hermaphrodite in left lateral view. (B) Surface of lip region of hermaphrodite. (C) Stomatal region of hermaphrodite in left lateral view. (D) Anterior part of dauer juvenile in left lateral view. (E) Surface of lip region of dauer juvenile. (F) Anterior gonad of mature hermaphrodite in right lateral view. (G) Anterior gonad of young hermaphrodite in right lateral view. (H) Tail region of hermaphrodite in left lateral view. (I) Male tail region in ventral view. (J) Male tail region in right lateral view. (K) Spicule and gubernaculum in right lateral view.

Collections of *T. tufae* from presumably undisturbed sites in California and of *T. atripennis* n. sp. from beetles endemic to East Asia suggest the simple interpretation that these two nematode species are native to those regions, respectively. However, the ancestral range of *T. tauri* n. sp. is still uncertain. Because sampling efforts in Central Europe have not, to our knowledge, revealed a nematode species typologically similar to *Tokorhabditis* sp., we speculate that *T. tauri* n. sp. originated outside of Europe and thus associated with other species of dung beetles prior to its first contact with *O. taurus*. The apparent nonspecificity of many dung-inhabiting nematodes with respect to dung-beetle hosts makes this scenario plausible (Sachs, 1950; Sudhaus et al., 1988). Even by this scenario, however, an ancestral range for *T. tauri* n. sp. outside of North America cannot be ruled out. First, populations of *O. taurus* throughout the eastern United States descend from founders that were accidentally introduced from an unknown source population, which was not necessarily in the beetles’ ancestral range (Hoebecke and Beucke, 1997; Rounds and Floate, 2012). Second, because *T. tauri* n. sp. also associates, for example, with *D. gazella*, which is native to sub-Saharan Africa, it is possible that the nematodes have been horizontally transmitted from other nonnative hosts in North American communities. Further sampling outside of the eastern United States, together with further work on the host beetles’ own biogeography, will help to reconstruct the history of *T. tauri* n. sp. in North America.

**Figure 11:**
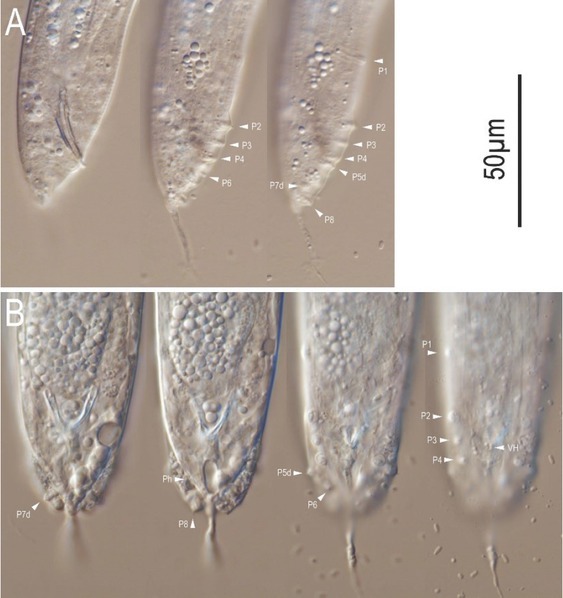
Differential interference contrast micrographs of the male tail region of *Tokorhabditis atripennis* n. sp. (A) Right lateral view in three focal planes. (B) Ventral view in four focal planes. Genital papillae are labeled with the prefix “P”; suffix “d” indicates papillae that open dorsally or laterally. Ph, phasmid.

With our report of a nondiplogastrid rhabditid from *Onthophagus* beetles, future studies can examine the interactions of a strictly microbivorous species with omnivorous ones, such as *D. monhysteroides*, in the same mesocosm. It is known that *D. monhysteroides* populations influence the bacterial and fungal species communities, including the ratio of bacterial to fungal abundance, in brood balls of *O. taurus* (Ledón-Rettig et al., 2018). Because *T. tauri* n. sp. may reach comparable densities in brood balls, it is possible – depending on the population dynamics of the two species – that *T. tauri* n. sp. either enhances or interferes with the effects of *D. monhysteroides*. Furthermore, manipulations of *T. tauri* n. sp. in brood balls can be measured with respect to a third trophic guild of nematodes consistently found on *O. taurus* and cow dung elsewhere: *Fictor* species, especially *Fictor* cf. *stercorarius* (Bovien, 1937) J. B. Goodey in T. Goodey, 1963 (Sachs 1950; Kühne 1996; E.J.R., unpubl.). Like *D. monhysteroides*, *Fictor* nematodes are omnivorous, although the latter are also predatory and often dimorphic in their feeding structures. Studies of other microhabitat systems have shown analogous interactions between multiple feeding guilds. For example, in sycamore figs, nematodes transmitted by fig wasps establish communities that change with the ontogeny of the fig. In this system, one of the transmitted diplogastrid species with polyphenism (i.e., having multiple feeding morphs) shows a higher frequency of its predatory forms when the nematode communities are mature, including more potential competitors for local resources (Susoy et al., 2016).

**Figure 12:**
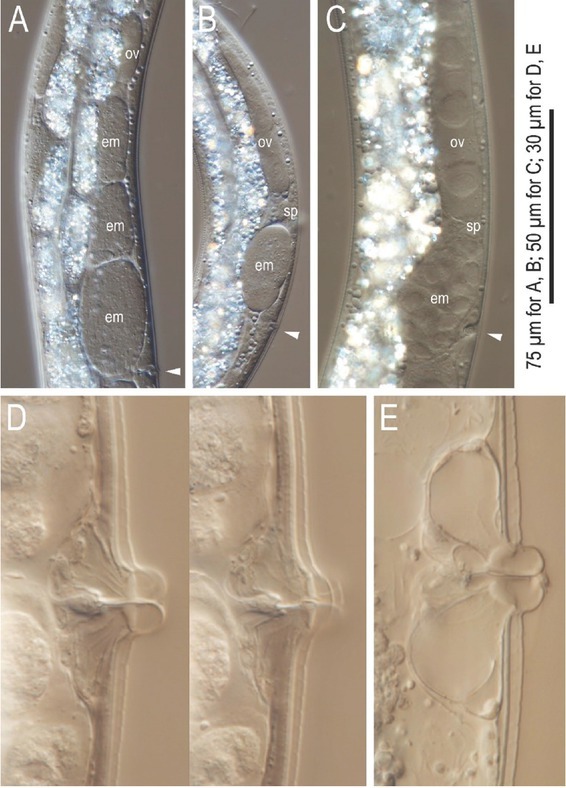
Differential interference contrast micrographs of the gonadal region of *Tokorhabditis atripennis* n. sp. hermaphrodites. All images are in right lateral view. (A)–(C) Anterior gonad of maturing adult (A), young adult (B), and fourth-stage juvenile (C). (D) Vulval region of young adult in two focal planes. (E) Vulval region of mature adult. em, embryos; ov, ovary; sp, sperm. Arrowhead marks vulva in (A)–(C).

**Figure 13:**
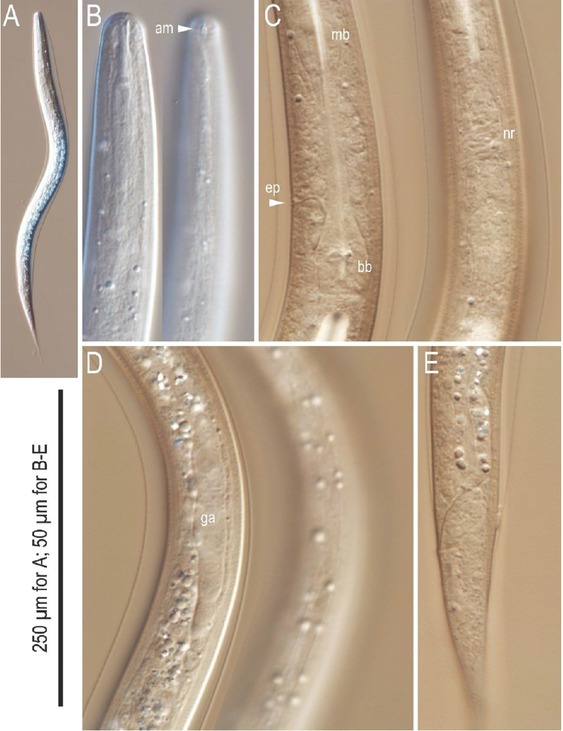
Differential interference contrast micrographs of the dauer juvenile of *Tokorhabditis atripennis* n. sp. All images in left lateral view. (A) Entire body. (B) Lip and anterior pharynx region in two focal planes. (C) Posterior pharynx region in two focal planes. (D) Mid-body region in two focal planes. (E) Anal region. am, amphid; bb, basal bulb; ep, excretory pore; ga, genital *anlage*; mb, median bulb; nr, nerve ring.

**Figure 14:**
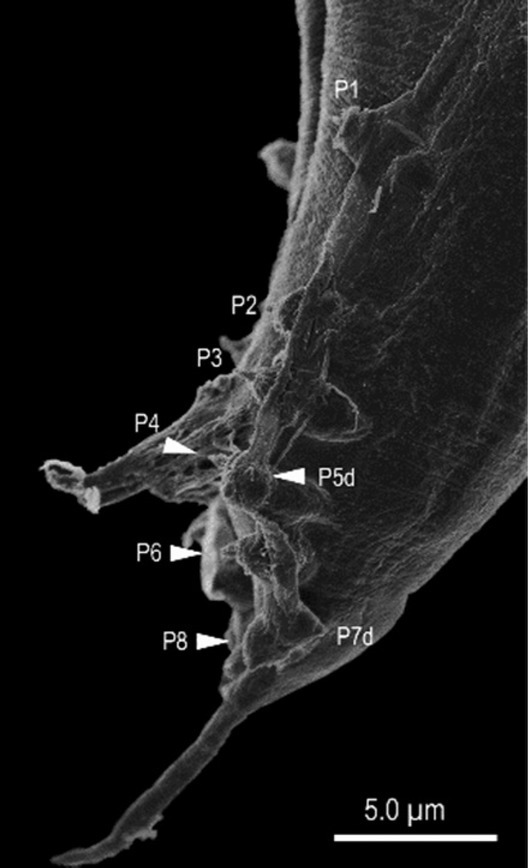
Scanning electron micrograph of the male tail of *Tokorhabditis atripennis* n. sp. Genital papillae are labeled with the prefix “P”; suffix “d” indicates papillae that open dorsally or laterally.

Likewise, a study of nematodes on the cadavers of killed cockchafer beetles (*Gymnogaster bupthalma*) showed that the presence of competing nematode species, in this case two diplogastrid species (*Acrostichus* sp. and *Pristionchus mayeri*), led to community change through increased induction of an alternative, predatory morph in *P. mayeri* and, in both populations, migration away from the community (Renahan and Sommer, 2021). With our report of *T. tauri* n. sp. here, we propose a system whereby different feeding guilds, including microbivores and two types of omnivores, can be manipulated to study their feedbacks with microbial communities and a developing insect host.

Beyond our report of *T. tauri* n. sp., our sampling efforts provide a route to phylogenetic comparisons of the phenomena we discuss above. The genus *Onthophagus* includes over 2,000 species worldwide, showing an unusual richness of its ecological contexts. Furthermore, the ability to rear multiple *Onthophagus* sp. in the laboratory makes experimental comparisons of their ecological interactions feasible (Rohner and Moczek, 2021). Our description of *T. atripennis* n. sp. from *Onthophagus atripennis* provides a comparator for potential interactions between *T. tauri* n. sp. and *O. taurus*. Given the presence of *T. tauri* n. sp. on the beetles *O. tuberculifrons* and *D. gazella* in the field, we predict that further sampling of other dung-burying beetles will broaden the comparative framework we propose.
